# Long non-coding RNA LINC01194 promotes the proliferation, migration and invasion of lung adenocarcinoma cells by targeting miR-641/SETD7 axis

**DOI:** 10.1186/s12935-020-01680-3

**Published:** 2020-12-07

**Authors:** Fanmei Meng, Yijing Zhou, Baohua Dong, Aiqin Dong, Jingtao Zhang

**Affiliations:** 1 Outpatient Department, Dongying District People’s Hospital, 333 Jinan Road, Dongying, 257085 Shandong China; 2 Department of Respiratory Medicine, Dongying District People’s Hospital, 333 Jinan Road, Dongying, Shandong China; 3 Internal Medicine-Neurology, Dongying District People’s Hospital, 333 Jinan Road, Dongying, Shandong China

**Keywords:** LINC01194, miR-641, SETD7, Lung adenocarcinoma

## Abstract

**Background:**

It is increasingly evidenced that long non-coding RNAs (lncRNAs) play an important role in various diseases. LncRNA LINC01194 acts as an oncogene in several cancer types. Nevertheless, the role of LINC01194 in lung adenocarcinoma (LUAD) has not yet been revealed.

**Methods:**

qRT-PCR was used to detect the expression of LINC01194, miR-641 and SETD7 mRNA, while western blot was exploited to examine SETD7 protein level. Cell proliferation was detected by colony formation and EdU assays. Transwell assays detected cell migration and invasion. TUNEL assay and flow cytometry analysis were used to detect cell apoptosis. RIP, RNA pull down and luciferase reporter assays detected the binding among LINC01194, miR-641 and SETD7.

**Results:**

LINC01194 was significantly upregulated in LUAD tissues and cell lines. Knockdown of LINC01194 resulted in decreased cell proliferation, migration and invasion, and increased apoptosis. Mechanistic experiments unveiled that LINC01194 augmented SETD7 expression in LUAD cells by competitively interacting with miR-641. Rescue experiments showed that miR-641 inhibition and SETD7 overexpression rescued the repressing impacts on LUAD cell proliferation, migration and invasion caused by LINC01194 knockdown.

**Conclusion:**

LINC01194 promotes the progression of LUAD by enhancing miR-641-targeted SETD7. The LINC01194/miR-641/SETD7 axis might provide new molecular targets for treating LUAD.

## Background

As we all know, lung cancer results in the most deaths among cancers in the worldwide. Based on previous studies, lung cancer mainly includes two types: non-small-cell lung cancer (NSCLC) and small cell lung cancer (SCLC). Specially, NSCLS accounts for 85% of all lung cancer cases [1]. In addition, NSCLS can be divided into lung squamous carcinoma (LUSC), lung adenocarcinoma (LUAD) and large cell carcinoma (LCC) [2, 3]. The role of many lncRNAs in LUSC and LUAD has been studied in previous researches [4, 5]. Here, we mainly focus on probing molecules involved in LUAD in this investigation.

Long non-coding RNAs (lncRNAs) are non-coding RNAs longer than 200 nucleotides and play a vital role in the progression of human diseases [6, 7]. Besides, reports also demonstrated that lncRNAs can work in certain cancer types [8, 9]. For instance, lncRNA OIP5-AS1 is apt to aggravate LUAD progression through regulating miR-448/Bcl-2 pathway [10]. Dysregulation of lncRNA-ATB has been shown to contribute to cell proliferation, migration and invasion, as well as EMT in cancers [11]. LncRNA LOC389641 promotes the progression of pancreatic ductal adenocarcinoma by regulating E-cadherin [12]. LncRNA XLOC_009167 becomes a novel biomarker owing to its overexpression in lung cancer [13]. There are countless examples about the lncRNAs that we can’t give them one by one. As for long intergenic non-protein coding RNA 1194 (LINC01194) that is mentioned in this article, it has been recently reported to have an important influence on colorectal carcinoma development [14]. However, the function of LINC01194 in LUAD still keeps unknown by the public, so there is urgent need to explore it.

In this research, we chiefly utilized diverse assays to verify that whether LINC01194 could act on the proliferation, migration, invasion of LUAD cells, such as colony formation assay, EdU assay, transwell assay, etc. Moreover, we further studied the interaction among LINC01194, miR-641 and SET domain containing 7, histone lysine methyltransferase (SETD7).

## Methods

### Clinical tissues

This study was implemented with the approval of the Ethics Committee of Dongying District People’s Hospital. Total of 64 pairs of LUAD tissues and adjacent non-tumor tissues were acquired from LUAD patients in the hospital of Dongying District People’s Hospital. Before surgery, the patients involved in this research received no any treatment and all of them signed the informed consents. Samples were processed with liquid nitrogen immediately after excision, and then maintained at -80℃ until subsequent use.

### Cell lines

The normal human bronchial epithelioid cell line (16HBE) and LUAD cell lines (A549, PC-9, HCC827, NCI-H1975 and NCI-H1299) in this study were attained from ATCC (Manassas, VA). In a humidified incubator with 5% CO_2_ at 37 °C, cells were cultivated in DMEM (Gibco, Rockville, MD) with additional 1% penicillin/streptavidin (Gibco) and 10% FBS (Gibco).

### Total RNA extraction and qRT-PCR

Total RNA extraction was performed by use of TRIzol Reagent (Invitrogen, Carlsbad CA), and then subjected to cDNA synthesis as per the protocol of PrimeScript Reverse Transcriptase Kit (Takara, Shiga, Japan). SYBR Green PCR Kit (Takara) was acquired for quantitative analyses. Relative expression of all target genes was processed based on 2^−ΔΔCt^ method and standardized to U6 snRNA or GAPDH mRNA.

### Western blot

Proteins extracted from LUAD cells were subjected to separation via 12% SDS-PAGE, followed by transferring onto PVDF membranes. After treating with 5% nonfat milk, the membranes were processed at 4℃ with the overnight incubation of primary antibodies (Abcam, Cambridge, MA) specific to SETD7 or GAPDH (loading control), which were further probed by the corresponding secondary antibodies at room temperature for 2 h. Finally, bands in the membranes were visualized with the enhanced chemiluminescence (ECL) detection system (Bio-Rad lab, Hercules, CA).

### Cell transfection

The shRNAs specific to LINC01194 or SETD7, and relative control-shRNAs, were all bought from GenePharma (Shanghai, China). The pcDNA3.1 vector (Invitrogen) was loaded with the cDNA sequence of SETD7 to acquire pcDNA3.1/SETD7, and the empty vector was exploited as the negative control. Besides, miR-641 mimics/inhibitor or NC mimics/inhibitor were produced by Ribobio (Guangzhou, China). A549 and HCC827 cells were transfected with indicated plasmids for 48 h with the help of Lipofectamine 3000 (Invitrogen).

### Colony formation assay

After 48 h of transfection, the processed LUAD cells were added to 6-well plates with 1000 cells per well, for the 14-day of cell culture purposes. Following fixation by formaldehyde, the colonies were stained by 0.5% crystal violet solution for counting manually.

### EdU assay

EdU assay was performed in processed LUAD cells in light of the protocol of BeyoClick™ EdU Cell Proliferation Kit (Beyotime, Shanghai, China). The EdU medium diluent was added to cell samples for 2 h, and then washed in PBS and subjected to DAPI staining. All cells were studied by an inverted microscope (Olympus, Tokyo, Japan).

### Transwell assay

Transwell chamber was pre-coated with Matrigel (BD Biosciences, Franklin Lakes, NJ) for invasion assay, and migration assay was performed similarly without Matrigel coating. 1 × 10^5^ LUAD cells were collected after transfection and cultured in serum-free medium after adding into the upper chamber. The complete medium was added to the lower chamber. 24 h later, cells in the lower chamber were fixed and stained in crystal violet solution for observation using microscope (Olympus).

### TUNEL assay

The transfected LUAD cell samples were rinsed utilizing PBS for fixing. Then, the TUNEL assay reagent (Merck KGaA, Darmstadt, Germany) was used for staining the apoptotic cells. After DAPI staining of cell nuclei, the optical microscopy (Olympus) was applied for analysis.

### Flow cytometry analysis

The apoptosis of LUAD cells was also determined using flow cytometry (BD Biosciences, Franklin Lakes, NJ). The double Annexin V/PI staining method was employed as required by the provider (Invitrogen). After double-staining in Binding Buffer, samples were analysis via flow cytometry.

### Subcellular fractionation

Subcellular fractionation assay in LUAD cells was accomplished with the application of Cytoplasmic & Nuclear RNA Purification Kit as per user manual (Norgen, Belmont, CA). LINC01194 content in cell nucleus and cell cytoplasm was separately detected using qRT-PCR, with U6 as the nuclear index and GAPDH as the cytoplasmic index.

### FISH

LUAD cells were washed in PBS after fixing, and then digested and air-dried. After culturing with LINC01194-specific FISH probe (Ribobio) in hybridization buffer, cell samples were treated with Hoechst staining and finally imaged under Olympus fluorescence microscope.

### RNA immunoprecipitation (RIP)

By applying Magna RIP™ RNA-Binding Protein Immunoprecipitation Kit (Millipore, Bedford, MA), RIP assay was conducted with anti-Ago2 antibody and anti-IgG antibody (negative control). The cultured cells were lysed in RIP lysis buffer, and then the lysates were treated with RIP buffer covering antibody-bound magnetic beads, followed by analysis of the precipitated RNAs by qRT-PCR.

### RNA pull down assay

The miR-641 sequences covering LINC01194 or SETD7 target sites (wild-type and mutant-type) were biotin-tagged into Bio-miR-641-WT/Mut probes. Cell lysates were mixed with probes and beads overnight, and then the retrieved RNA mixture was monitored by qRT-PCR.

### Luciferase reporter assay

The LINC01194 or SETD7 3′UTR fragments containing miR-641 target sites (wild-type and mutant-type) were inserted to pmirGLO dual-luciferase reporter vectors (Promega, Madison, WI), which were then named as LINC01194-WT/Mut and SETD7 3′UTR-WT/Mut. The vectors were co-transfected with miR-641 mimics or NC mimics into LUAD cells. Their luciferase intensity was individually estimated using dual-luciferase reporter assay system (Promega).

### Statistical analyses

Data were exhibited as means ± SD of more than two independently conducted assays. All group differences were compared in form of Student’s t-test or one-way analysis of variance (ANOVA), by applying GraphPad Prism 7 (La Jolla, CA). The experimental results were thought as statistically significant when p < 0.05.

## Results

### LINC01194 is given to high expression and it promotes malignant processes in LUAD

**Fig. 1:**
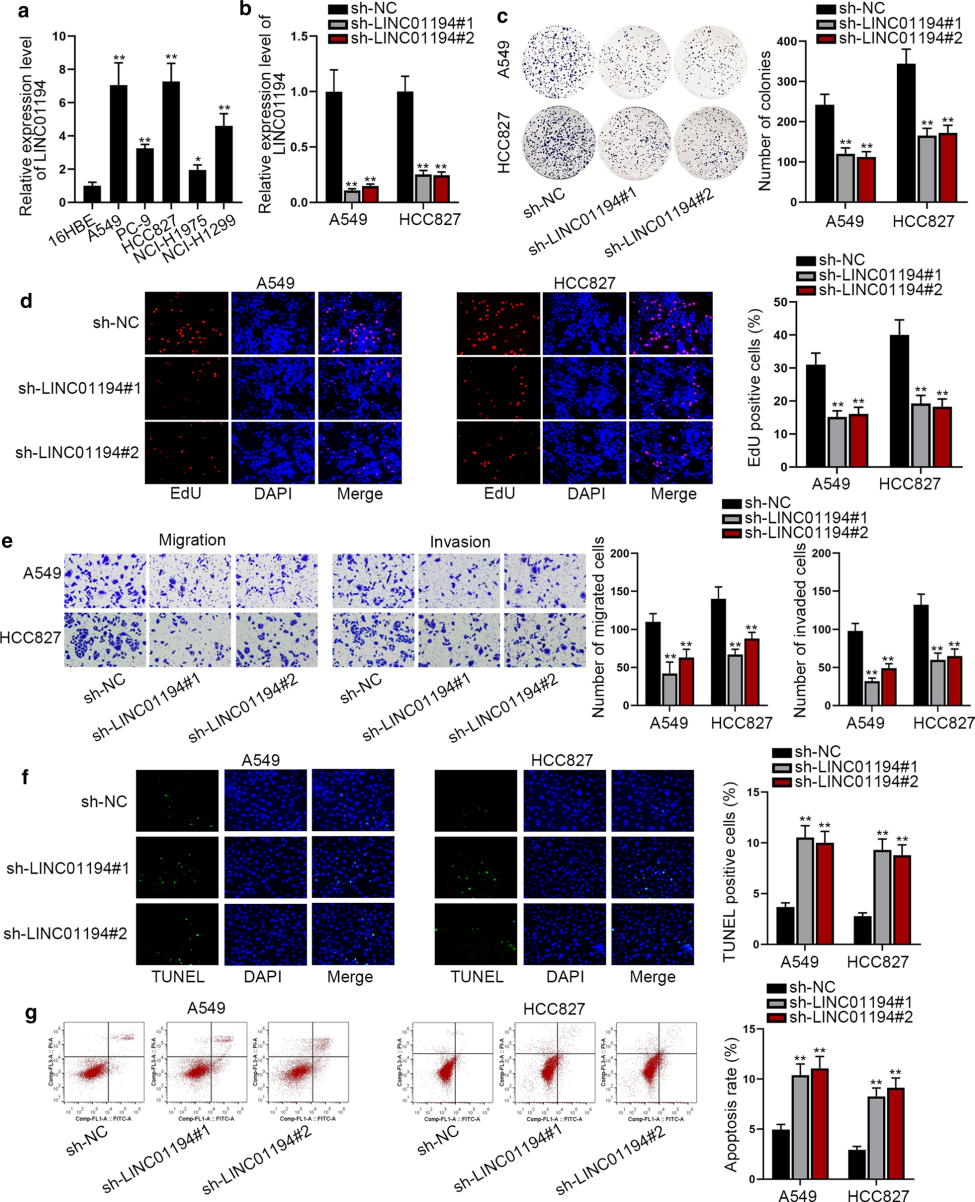
LncRNA LINC01194 is given to high expression and promotes malignancy in LUAD. **a** LINC01194 expression was detected by qRT-PCR in normal human bronchial epithelioid cell line (16HBE) and LUAD cell lines (A549, PC-9, HCC827, NCI-H1975 and NCI-H1299). **b** qRT-PCR was applied to access the interference efficiency of LINC01194 in A549 and HCC827 cells. **c**, **d** Colony formation assay and EdU assay showed that down-regulation of LINC01194 significantly decreased LUAD cell proliferation. **e** Transwell assay was utilized to detect the ability of LUAD cells migration and invasion. **f**, **g** The apoptosis of LUAD cells was accessed by TUNEL assay and flow cytometry analysis. All results were displayed as the mean ± SD, n = 3, Student’s t test or one-way analysis of variance was carried out for evaluating the differences between groups. *P < 0.05, **P < 0.01

In order to probe the function of LINC01194 in LUAD in detail, we primarily examined LINC01194 expression in normal and malignant settings. Notably, LINC01194 presented high expression in 64 LUAD samples relative to adjacent non-cancerous controls (Additional file 1: Figure S1A). Consistently, the results of qRT-PCR analysis showed that LINC01194 expressed much higher in LUAD cell lines (A549, PC-9, HCC827, NCI-H1975 and NCI-H1299) than in the normal human bronchial epithelioid cell line 16HBE, particularly in A549 and HCC827 cells (Fig. 1a). On this basis, we selected A549 and HCC827 cells for the following investigation. Then we used qRT-PCR to access the interference efficiency of LINC01194 after transfecting sh-LINC01194#1 or sh-LINC01194#2 into A549 and HCC827 cells. The results demonstrated that the expression of LINC01194 was obviously cut down in LUAD cells after the transfection with sh-LINC01194#1/2in comparison to those with sh-NC control (Fig. 1b). To further verify whether LINC01194 takes effect on the LUAD cell proliferation, apoptosis, migration and invasion, the loss-of-function assays were carried out. The results of colony formation assay and EdU assay presented that the number of colonies and the rate of EdU stained positive cells were both declined markedly after transfecting sh-LINC01194#1 and sh- LINC01194#2 into A549 and HCC827 cells (Fig. 1c, d), which explained that down-regulation of LINC01194 blocked LUAD cell proliferation. Besides, the consequence of transwell assay showed that the capacities of cell migration and invasion were also significantly decreased after intervening LINC01194 (Fig. 1e). In addition to assessing the abilities of cell proliferation, migration and invasion, it was essential to analyze the apoptosis of LUAD cells under the same circumstances. Hence, we conducted TUNEL assay and flow cytometry analysis. Results demonstrated that the percentage of TUNEL positive cells and cell apoptosis rate were both elevated due to LINC01194 interference (Fig. 1f, g), which expressed that LINC01194 silencing promoted LUAD cell apoptosis. Based on above investigations, we concluded that LINC01194 is overexpressed in LUAD cells and it plays an impellent role in LUAD.

### LINC01194 is capable to combine with miR-641

**Fig. 2:**
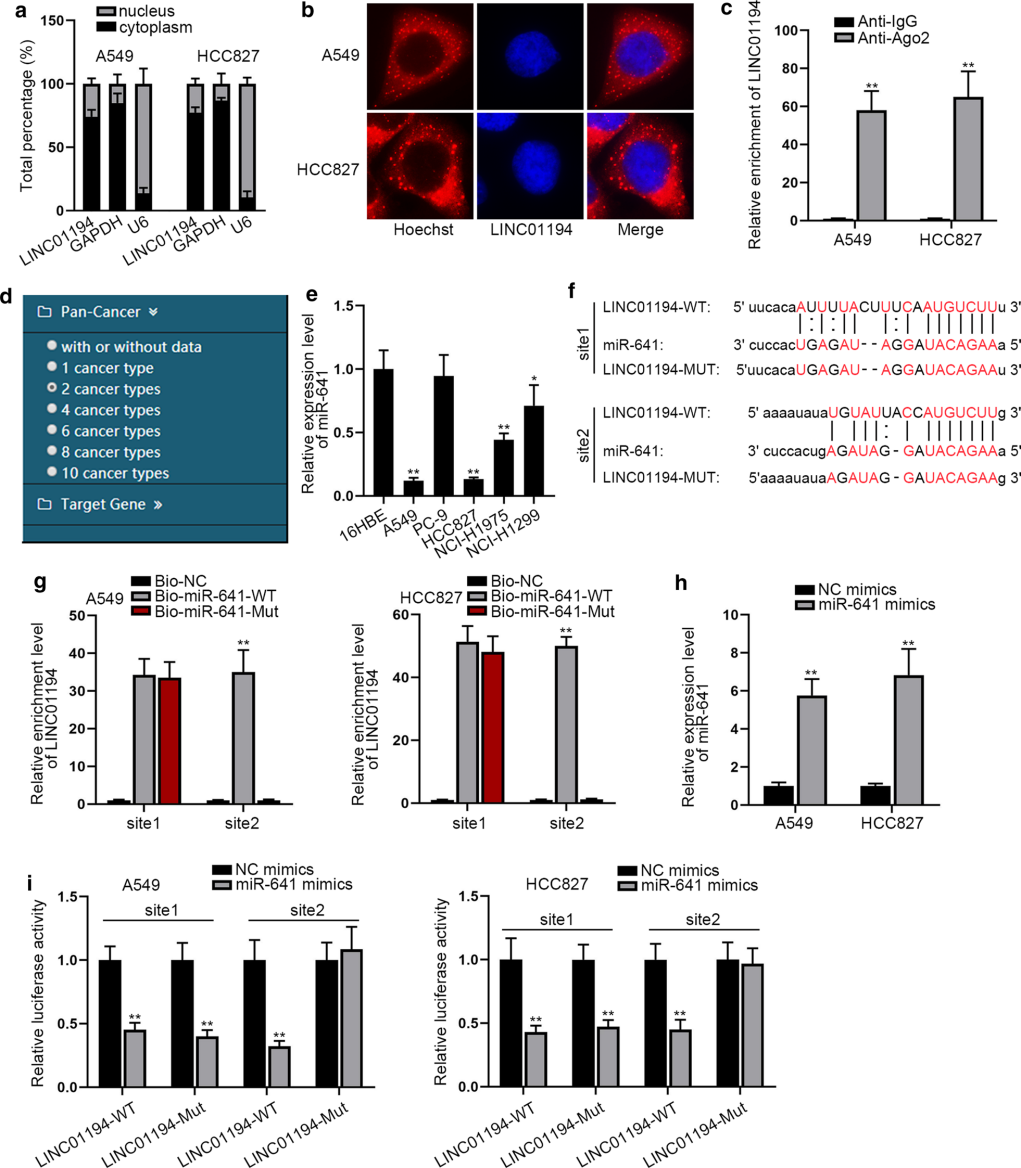
LINC01194 is capable to combine with miR-641. **a**, **b** The nuclear/cytoplasmic separation assay and FISH assay was used to confirm the subcellular localization of LINC01194 in LUAD cells. **c** RIP was applied to detect the enrichment of LINC01194 by anti-Ago2 or anti-IgG. **d** The screening condition of LINC01194-interacting miRNAs by starBase v2.0. **e** qRT-PCR analysis was conducted to detect the overexpression efficiency of miR-461. **f** The potential binding sites between LINC01194 and miR-641 were hypothesized by starBase v2.0. **g** RNA pull down assay confirmed the binding of miR-641 to the site 2 of LINC01194. **h** qRT-PCR analysis was conducted to detect the overexpression efficiency of miR-641 in two LUAD cells. **i** Luciferase reporter assays were used to confirm the interaction between LINC01194 and miR-641. All results were displayed as the mean ± SD, n = 3, Student’s t test or one-way analysis of variance was carried out for evaluating the differences between groups. *P < 0.05, **P < 0.01

To know the regulatory mechanism of LINC01194 in LUAD, we then tested its subcellular localization. By the outcomes of nuclear/cytoplasmic separation assay and FISH assay, we found that most LINC01194 was distributed in the cytoplasm (Fig. 2a, b). The results of RIP assay showed that LINC01194 was enriched in Anti-Ago2 groups but not in Anti-IgG groups (Fig. 2c), which suggested that LINC00313 might play a role through ceRNA regulation mode. Hence, we utilized starBase software to screen potential miRNAs interacting with LINC01194, and then selected miR-641 under the condition of two cancer types (Fig. 2d). Subsequently, we unveiled that the expression of miR-641 was lower in five LUAD cell lines than that in16HBE cells (Fig. 2e). Besides, the low expression trend of miR-641 was also certified in 64 LUAD specimens compared to matched non-tumor ones (Additional file 1: Figure S1B). Then, starBase v2.0 software (http://starbase.sysu.edu.cn/) showed that there are two binding sites between LINC01194 and miR-641 (Fig. 2f). Based on above data, we guessed that miR-641 was the downstream of LINC01194 in LUAD. To testify this, we continued with the following experiments. The data from RNA pull down assay exhibited that LINC01194 could be undoubtedly pulled down by Bio-miR-641-WT, while such phenomena no more occurred when site 2 was mutated (Fig. 2g). Then, qRT-PCR verified that after transfecting miR-641 mimics into both LUAD cells, the relative expression of miR-641 was markedly up-regulated (Fig. 2h). Further, we carried out the luciferase reporter assay to validate the interaction relationship between LINC01194 and miR-641. The results indicated that the luciferase activity of wild-type and site 1-mutated LINC01194 was significantly cut down by miR-641 mimics, whereas that of site 2-mutated LINC01194 was nearly unchanged (Fig. 2i), further confirming that miR-641 bound with LINC01164 at site 2. In summary, LINC01194 interacts with miR-641 in LUAD cells.

### Overexpression of miR-641 suppresses effectively the progression of LUAD

**Fig. 3:**
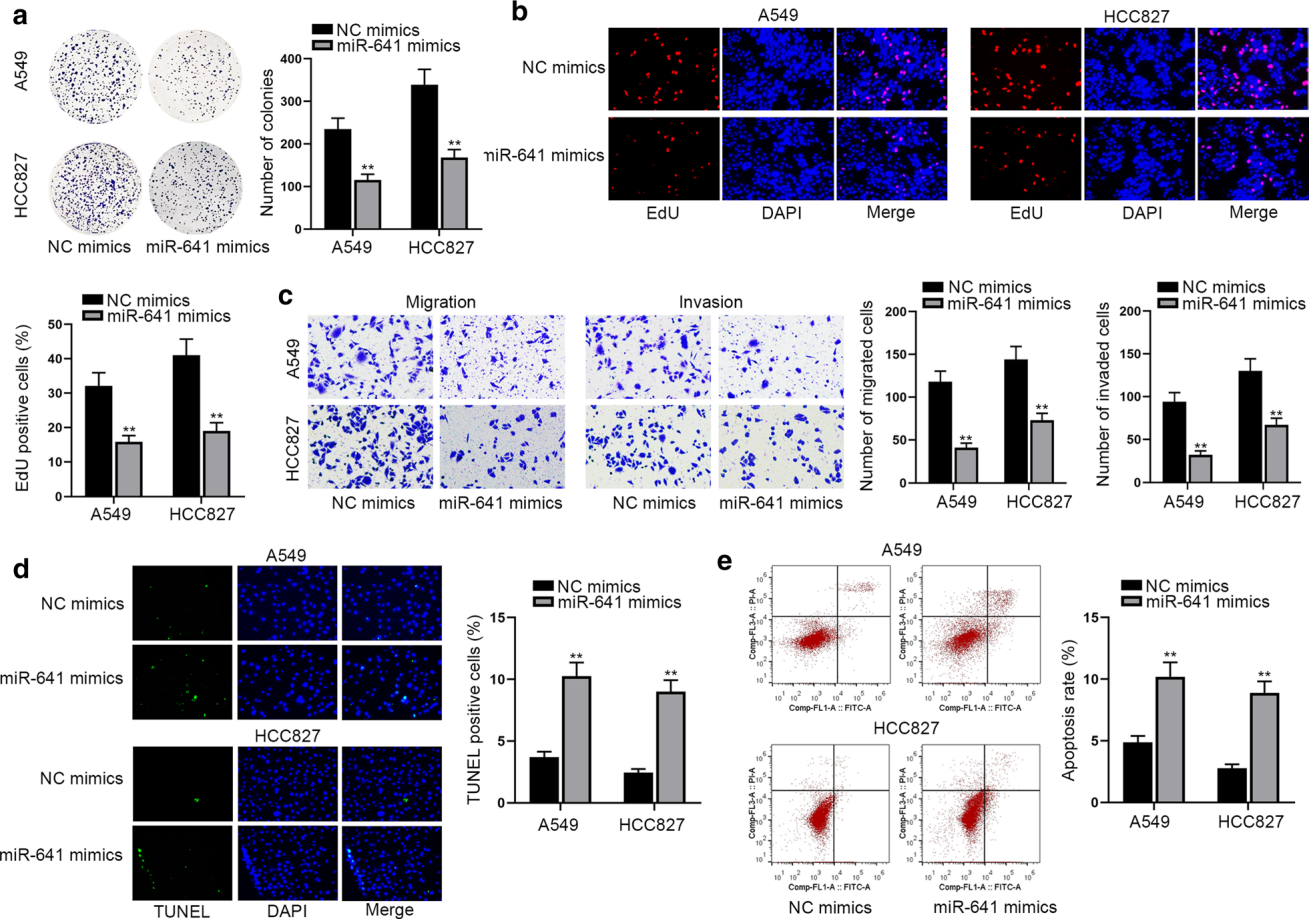
Overexpression of miR-641 suppresses effectively the progression of LUAD. **a**, **b** The capacity of cell proliferation was probed by colony formation assay and EdU assay. **c** The transwell assay was used to measure cell migratory and invasive abilities. **d**, **e** TUNEL assay and flow cytometry analysis were applied to analyze cell apoptosis. All results were displayed as the mean ± SD, n = 3, comparisons between groups were subjected to Student’s t test. **P < 0.01

In order to further detect the effect of miR-641 on the progression of LUAD, we adopted gain-of-function assays. The results of colony formation assay and EdU assay presented that overexpression of miR-641 effectively suppressed the proliferation ability of LUAD cells (Fig. 3a, b). Furthermore, the results of transwell assay displayed that miR-641 up-regulation impaired the migration and invasion ability of LUAD cells as well (Fig. 3c). In the end, we utilized TUNEL assay and flow cytometry analysis to detect the change in LUAD cell apoptosis. It showed that up-regulation of miR-641 augmented the rates of TUNEL positive cells and cell apoptosis, indicating that miR-641 increasing promoted LUAD cell apoptosis (Fig. 3d, e). Taken together, miR-641 hinders the development of LUAD.

### LINC01194 serves as a ceRNA to regulate SETD7 by sponging miR-641

**Fig. 4:**
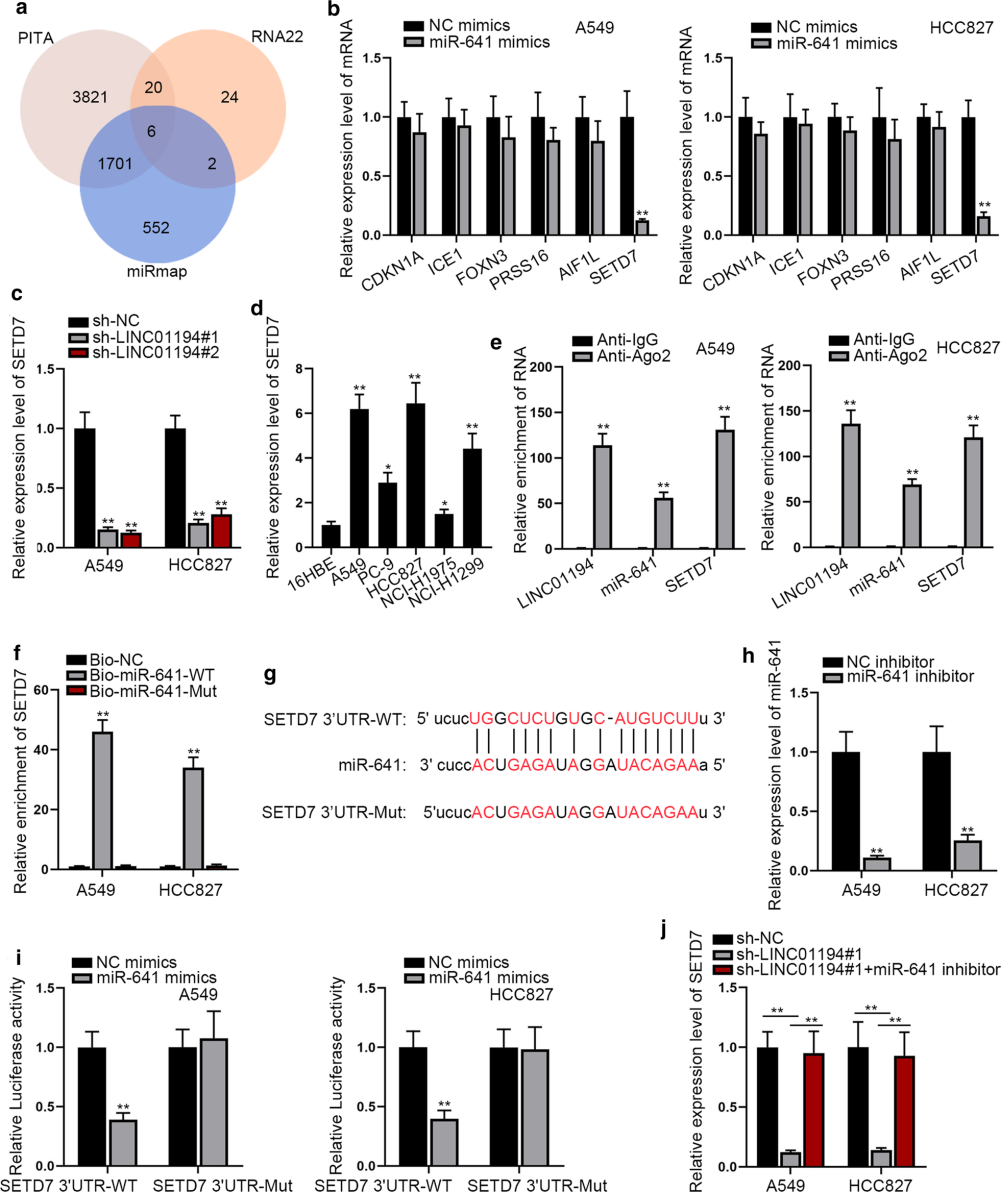
LINC01194 serves as a ceRNA to elevate the expression of SETD7 by competing for miR-641. **a** StarBase v2.0 screened out 6 targets of miR-641 that shared by PITA, miRmap and RNA22 tools. **b** The influences of miR-641 on above selected 6 gens were detected by qRT-PCR. **c** The qRT-PCR was used to analyze the expression of SETD7 after transfection with sh-LINC01194#1&2. **d** The expression of SETD7 in LUAD cell lines relative to the normal 16HBE cells was tested by qRT-PCR. **e**, **f** RIP and pull down assays to detect three RNAs whether co-exist in RISC and combined each other. **g** StarBase v2.0 predicted the possible binding sites between miR-641 and SETD7. **h** qRT-PCR was used to access the interference efficiency of miR-641. **i** Luciferase reporter assay was used to confirm the interaction between miR-641 and SETD7. **j** SETD7 expression in indicated LUAD cells was examined via qRT-PCR. All results were displayed as the mean ± SD, n = 3, comparisons between groups were subjected to Student’s t test or one-way ANOVA. **P < 0.01

To find the target genes of miR-641, we filtered 6 mRNAs that could combine with miR-641 through starBase (Fig. 4a). Next, qRT-PCR was performed to detect the expression of above 6 genes in LUCD cells after transfecting with miR-641 mimics. The results exhibited that only the level of SETD7 was obviously lowered by miR-641 upregulation, while that of the remaining 5 mRNAs (CDKN1A, ICE1, FOXN3, PRSS1 and AIF1L) was not (Fig. 4b). Besides, we revealed that SETD7 expression was decreased markedly owing to down-regulation of LINC01194 (Fig. 4c and Additional file 1: Figure S1C). Based on above data, SETD7 was chosen to be the subject for following study. Thereafter, it was unveiled that SETD7 had significant higher expression in LUAD tissues than paired non-tumor tissues (Additional file 1: Figure S1D), so did in LUAD cell lines than in 16HBE cells (Fig. 4d). The RIP assay data explained that LINC01194, miR-641 and SETD7 were enriched in Anti-Ago2 group but not in Anti-IgG groups (Fig. 4e), manifesting that LINC01194, miR-641 and SETD7 were co-existed in RNA-induced silencing complex (RISC). In the meantime, the outcomes of RNA pull down assay verified the strong enrichment of SETD7 in Bio-miR-641-WT group but not the mutant group (Fig. 4f). Additionally, starBase software was used to predict the binding site between miR-641 and SETD7 3′UTR, and the sequence of SETD7 3′UTR-Mut that could no longer combine with miR-641 was obtained (Fig. 4g). To explore the biological connection between miR-641 and SETD7, miR-641 was stably knocked down in LUAD cells by the transfection of miR-641 inhibitor (Fig. 4h). According to luciferase reporter assay results, the luciferase activity of SETD7-WT was significantly lessened by enhanced miR-641, while no change was observed in that of SETD7 3′UTR-Mut under same contexts (Fig. 4i). Of note, we discovered that the declined expression of SETD7 owing to LINC01194 knockdown was recovered under the inhibition of miR-641 (Fig. 4j and Additional file 1: Figure S1E). Taken together, LINC01194 positively modulates SETD7 expression by competitively binding to miR-641.

### Down-regulation of SETD7 restrains the oncogenic phenotypes of LUAD cells

**Fig. 5:**
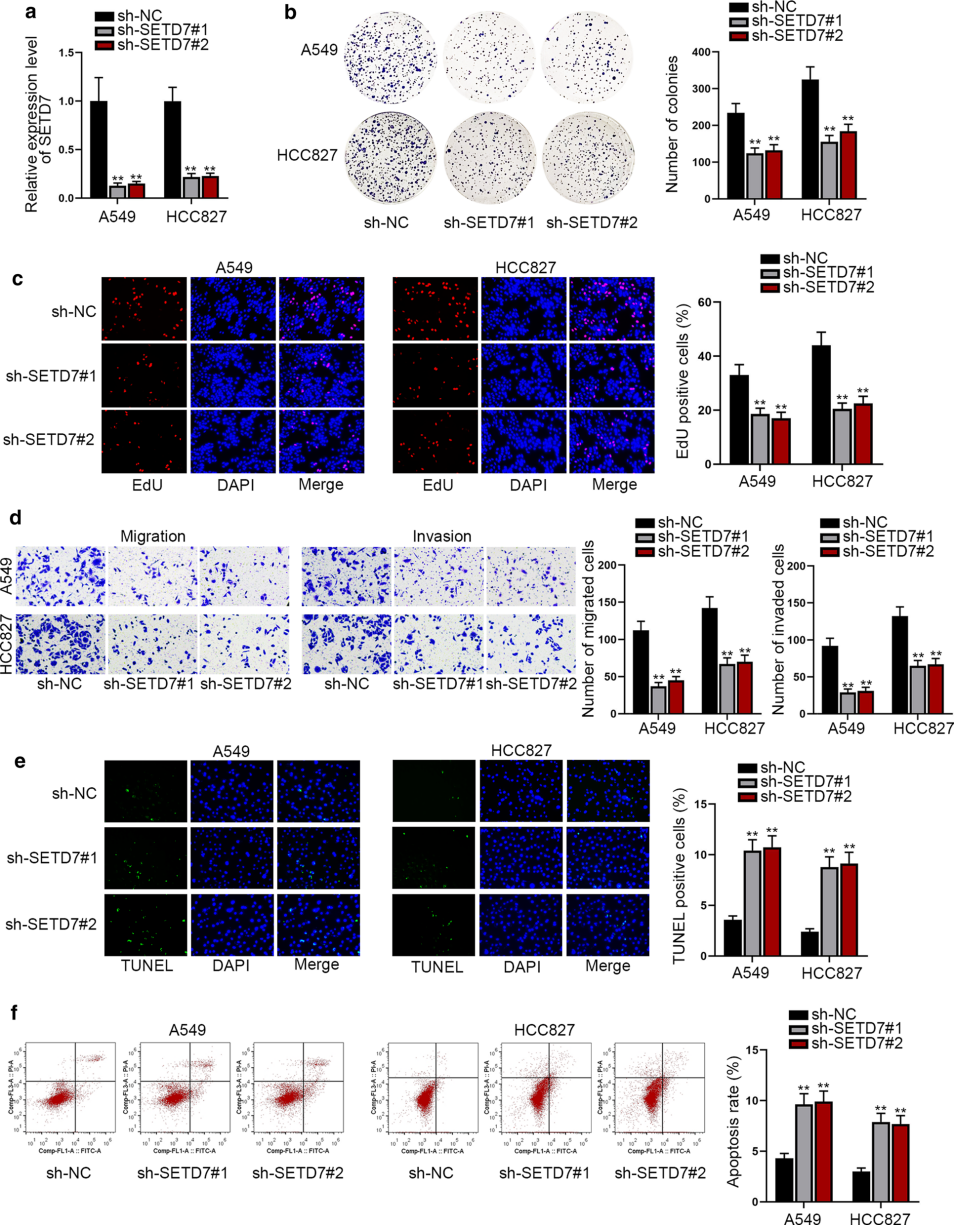
Down-regulation of SETD7 suppresses the oncogenic behaviors of LUAD cells. **a** The interference efficiency of SETD7 was assessed by qRT-PCR. **b**, **c** Colony formation assay and EdU assay showed that down-regulation of SETD7 significantly decreased cell proliferation in LUAD. **d** The transwell assay was utilized to test the migration and invasion ability of LUAD cells. **e**, **f** The apoptosis of LUAD cells were evaluated by TUNEL assay and flow cytometry analysis. All results were displayed as the mean ± SD, n = 3, and the comparisons between groups were analyzed via one-way ANOVA. **P < 0.01

To further confirm the function of SETD7 in LUAD, we first down-regulated the expression of SETD7 by transfecting sh-SETD7#1 or sh-SETD7#2 into A549 and HCC827 cells (Fig. 5a and Additional file 1: Figure S1F). Then we performed colony formation assay and EdU assay to detect the LUAD cell proliferation, transwell assay to evaluate cell migration and invasion, and TUNEL assay and flow cytometry analysis to assess the LUAD cell apoptosis. The results of colony formation assay and EdU assay indicated that the proliferative ability of LUAD cells were distinctly decreased under SETD7 deficiency (Fig. 5b, c). Besides, transwell assay data exhibited that the abilities of LUAD cells to migrate and invade were also cut down due to the absence of SETD7 (Fig. 5d). Finally, depending on the results of TUNEL assay and flow cytometry analysis, we unmasked that knockdown of SETD7 augmented the rate of LUAD cell apoptosis (Fig. 5e, f). To sum up, SETD7 drives the progression of LUAD.

### LINC01194 activates LUAD progression through targeting miR-641/SETD7 axis

**Fig. 6:**
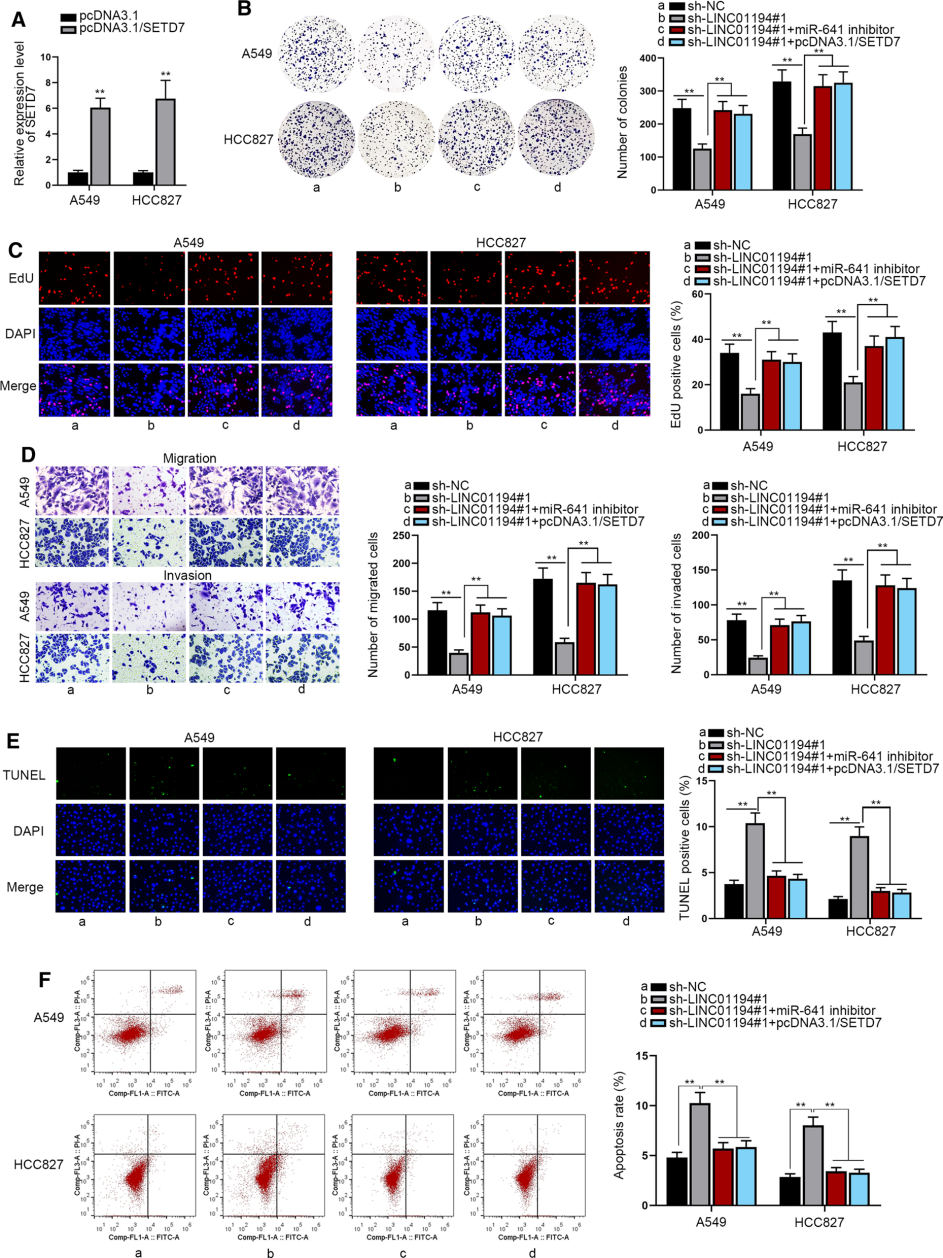
LINC01194 activates LUAD progression via miR-641/SETD7 axis. **a** The overexpression efficiency of SETD7 was tested by qRT-PCR. **b**, **c** The capacity of cell proliferation was examined by colony formation assay and EdU assay. **d** Cell migratory and invasive abilities were measured by transwell assays. **e**, **f** TUNEL assay and flow cytometry analysis were applied to analyze cell apoptosis. All results were displayed as the mean ± SD, n = 3, comparisons between groups were subjected to Student’s t test or one-way ANOVA. **P < 0.01

In the last step, we performed a string of recuse assays to further confirm the function of LINC01194/miR-641/SETD7 signaling in LUAD. Prior to that, we validated that SETD7 was sharply up-regulated at both mRNA and protein levels by transfecting pcDNA3.1/SETD7 into two LUAD cells (Fig. 6a and Additional file 1: Figure S1G). We then assessed the impact of miR-641 inhibition or SETD7 overexpression on the function of LINC01194-depleted cells. The results of colony formation assay and EdU assay displayed that loss of LINC01194 caused repression on LUAD cell proliferation was completely offset by inhibited miR-641 and enhanced SETD7 (Fig. 6b, c). As demonstrated in Fig. 6d, decreasing of miR-641 and SETD7 up-regulation fully counteracted the suppressive influence of SNHG16 silence on LUAD cell migration and invasion. In addition, miR-641 decreasing and SETD7 up-regulation also fully recovered the promoting effect of down-regulated LINC01194 on LUAD cell apoptosis (Fig. 6e, f). Collectively, our data revealed that LINC01194 accelerates the progression of LUAD by targeting miR-641/SETD7 axis.

## Discussion

It has been reported that some lncRNAs are chosen to be candidate targets because of their functional roles in LUAD. For example, since the smaller tumor size is connecting to DGCR5 down-regulation [15]. LncRNA RCC2 promotes significantly LUAD cell migration, invasion, and proliferation [16]. SBF2-AS1 contributes to the tumorigenesis of LUAD by sponging miR-338-3p and miR-362-3p to regulate E2F1 expression [17]. It is reported that HOXA11 functions as an oncogene in LUAD, and LUAD patients have great overall survival rates with down-regulated HOXA11 [18]. LPCAT1 has been reported to be involved in the progression, metastasis and recurrence of LUAD as well [19]. HCP5 can serve as a potential therapeutic target in LUAD because overexpression of HCP5 is positively correlated with the poor prognosis of LUAD patients [20]. Taken together, we can see it clearly that these lncRNAs make great differences to LUAD development.

As for this study, we aimed to analyze the expression level and role of LINC01194 and its molecular mechanism in LUAD. Previously, LINC01194 has been suggested to play oncogenic parts in several different cancer types, such as colorectal cancer [14] and laryngeal squamous cell carcinoma [21]. Importantly, a recent work also indicated LINC01194 as a carcinogene in NSCLC [22]. Consistent with these findings, here we discovered the upregulation of LINC01194 in LUAD tissues and cells, and that silencing LINC01194 hampered LUAD cell proliferation, migration and invasion. Based on these observations, we believe that LINC01194 may also be a promising therapeutic target for LUAD.

In addition, it is still necessary for us to probe the downstream regulation mode of LINC01194 in LUAD. Recently, a cytoplasmic lncRNA usually functions as a ceRNA to exert its post-transcriptional regulation on gene expression [23]. For instance, TTN-AS1 boosts CDK5 via sequestering miR-142-5p to facilitate malignancy in LUAD [24]. In the present work, we unveiled miR-641 as the downstream sponged by LINC01194. MiR-641 has been previously supported as a tumor suppressor in human malignancies including lung cancer [25, 26]. Here, we also validated that miR-641 was a tumor-inhibitor which was lowly-expressed in LUAD.

Furthermore, SETD7 was then recognized as the target of miR-641. SETD7 has been reported to play a tumor-contributing role in breast cancer [27] and hepatocellular carcinoma [28]. However, with respect to the role of SETD7 in lung cancer, there is a dispute over this matter in the current literatures. Cao et al. unveiled that SETD7 is downregulated in LUAD and has restraining effects on LUAD cell migration and invasion [29]. In contrast, Fu et al. argue that SETD7 activates Hedgehog pathway to aggravate the tumorigenesis of NSCLC [8], and Lezina et al. believe that SEDT7 contributes to cell proliferation in lung tumors [30]. In this work, we disclosed that SETD7 was upregulated in LUAD tissues and cells, and that the absence of SETD7 led to impaired proliferative, migratory and invasive capacities of LUAD cells. In other word, SETD7 was unveiled as a tumor-promoter in LUAD by the current research. Moreover, we certified that LINC01194 contributed to LUAD progression through miR-641/SETD7 signaling.

## Conclusion

In this investigation, LINC01194 is highly expressed in LUAD and is recognized as a carcinogenic gene to push LUAD cell proliferation, migration and invasion. In addition, our study also presents that miR-641 could combine with LINC01194 and down-regulation of miR-641 also elevates cell proliferation. In this work, we further identify that SETD7 is downstream gene of miR-641, and SETD7 increasing tends to promote LUAD cell progression as well. In conclusion, all data verify that LINC01194/miR-641/SETD7 regulatory axis contributes to LUAD progression, which implies that LINC01194 may serve as a biomarker and potential therapeutic target for LUAD.

## Supplementary Information

**Additional file 1: Figure S1.** A and B. The expression of LINC01194 and miR-641 in 64 LUAD tissues and paired non-tumor tissues was tested via qRT-PCR. C. Western blot detected the protein level of SETD7 in A549 and HCC827 cells with or without LINC01194 inhibition. D. The expression of SETD7 in 64 LUAD tissues and paired non-tumor tissues was tested via qRT-PCR. E, F and G. The level of SETD7 protein in two LUAD cells under diverse conditions was determined by western blot. **P < 0.01.

## Data Availability

Relevant data and materials have been shown within this manuscript and the additional files.

## Abbreviations

SETD7: SET domain containing 7, histone lysine methyltransferase
LINC01194: Long intergenic non-protein coding RNA 1194
LUAD: Lung adenocarcinoma
NSCLC: Non-small-cell lung cancer
SCLC: Small cell lung cancer
LUSC: Lung squamous carcinoma
LCC: Large cell carcinoma
lncRNAs: Long noncoding RNAs
miRNAs: MicroRNAs
mRNA: Messenger RNA
ceRNA: Competing endogenous RNA
WT: Wild-type
Mut: Mutant
EdU: 5-Ethynyl-20-deoxyuridine
TUNEL: The terminal deoxynucleotidyl transferase dUTP nick-end labelling
FISH: Fluorescent in situ hybridization
qRT-PCR: Quantitative real-time PCR
FBS: Fetal bovine serum
RIP: RNA immunoprecipitation
DMEM: Dulbecco’s Modified Eagle’s Medium
GAPDH: Glyceraldehyde-3-phosphate dehydrogenase
SD: Standard deviation
ANOVA: Analysis of variance
