# Supplementary figures and images for: Long non-coding RNA LINC01194 promotes the proliferation, migration and invasion of lung adenocarcinoma cells by targeting miR-641/SETD7 axis
Source: Cancer Cell Int. 2020 Dec 7;20:588. doi: 10.1186/s12935-020-01680-3 (PMC7722326; doi:10.1186/s12935-020-01680-3)

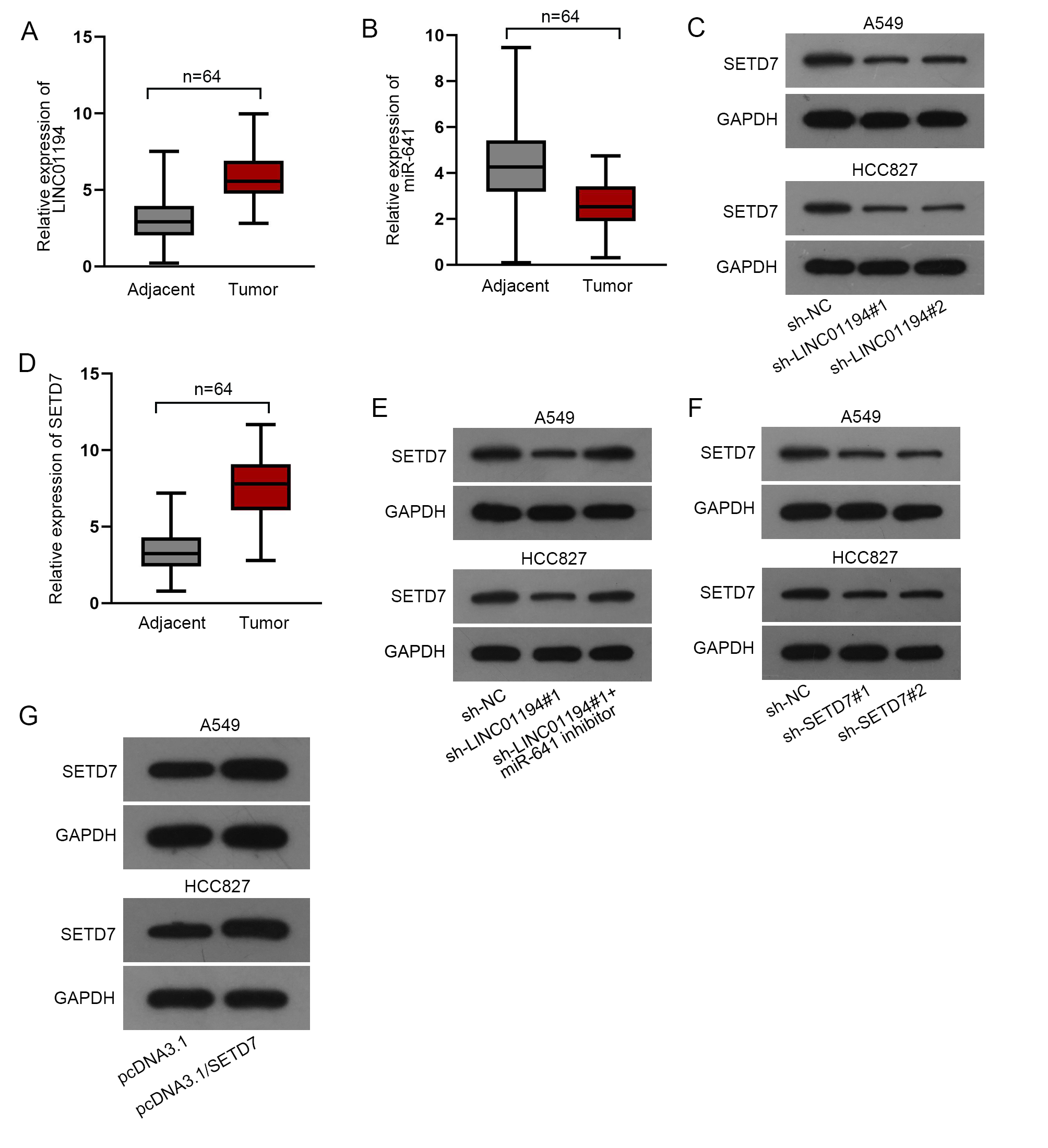

Supplement: Supplementary file 1 — Additional file 1: Figure S1. A and B. The expression of LINC01194 and miR-641 in 64 LUAD tissues and paired non-tumor tissues was tested via qRT-PCR. C. Western blot detected the protein level of SETD7 in A549 and HCC827 cells with or without LINC01194 inhibition. D. The expression of SETD7 in 64 LUAD tissues and paired non-tumor tissues was tested via qRT-PCR. E, F and G. The level of SETD7 protein in two LUAD cells under diverse conditions was determined by western blot. **P < 0.01. [file 12935_2020_1680_MOESM1_ESM.tif]
